# Electronic Health Record Usability, Satisfaction, and Burnout for Family Physicians

**DOI:** 10.1001/jamanetworkopen.2024.26956

**Published:** 2024-08-29

**Authors:** A. Jay Holmgren, Nathaniel Hendrix, Natalya Maisel, Jordan Everson, Andrew Bazemore, Lisa Rotenstein, Robert L. Phillips, Julia Adler-Milstein

**Affiliations:** 1 Division of Clinical Informatics and Digital Transformation, University of California, San Francisco; 2 American Board of Family Medicine, Center for Professionalism and Value in Health Care, Washington, DC; 3 Office of the National Coordinator for Health Information Technology, Department of Health and Human Services, Washington, DC

## Abstract

**Importance:**

Electronic health record (EHR) work has been associated with decreased physician well-being. Understanding the association between EHR usability and physician satisfaction and burnout, and whether team and technology strategies moderate this association, is critical to informing efforts to address EHR-associated physician burnout.

**Objectives:**

To measure family physician satisfaction with their EHR and EHR usability across functions and evaluate the association of EHR usability with satisfaction and burnout, as well as the moderating association of 4 team and technology EHR efficiency strategies.

**Design, Setting, and Participants:**

This study uses data from a cross-sectional survey conducted from December 12, 2021, to October 17, 2022, of all family physicians seeking American Board of Family Medicine recertification in 2022.

**Exposure:**

Physicians perceived EHR usability across 6 domains, as well as adoption of 4 EHR efficiency strategies: scribes, support from other staff, templated text, and voice recognition or transcription.

**Main Outcomes and Measures:**

Physician EHR satisfaction and frequency of experiencing burnout measured with a single survey item (“I feel burned out from my work”), with answers ranging from “never” to “every day.”

**Results:**

Of the 2067 physicians (1246 [60.3%] younger than 50 years; 1051 men [50.9%]; and 1729 [86.0%] practicing in an urban area) who responded to the survey, 562 (27.2%) were very satisfied and 775 (37.5%) were somewhat satisfied, while 346 (16.7%) were somewhat dissatisfied and 198 (9.6%) were very dissatisfied with their EHR. Readability of information had the highest usability, with 543 physicians (26.3%) rating it as excellent, while usefulness of alerts had the lowest usability, with 262 physicians (12.7%) rating it as excellent. In multivariable models, good or excellent usability for entering data (β = 0.09 [95% CI, 0.05-0.14]; *P* < .001), alignment with workflow processes (β = 0.11 [95% CI, 0.06-0.16]; *P* < .001), ease of finding information (β = 0.14 [95% CI, 0.09-0.19]; *P* < .001), and usefulness of alerts (β = 0.11 [95% CI, 0.06-0.16]; *P* < .001) were associated with physicians being very satisfied with their EHR. In addition, being very satisfied with the EHR was associated with reduced frequency of burnout (β = −0.64 [95% CI, −1.06 to −0.22]; *P* < .001). In moderation analysis, only physicians with highly usable EHRs saw improvements in satisfaction from adopting efficiency strategies.

**Conclusions and Relevance:**

In this survey study of physician EHR usability and satisfaction, approximately one-fourth of family physicians reported being very satisfied with their EHR, while another one-fourth reported being somewhat or very dissatisfied, a concerning finding amplified by the inverse association between EHR satisfaction and burnout. Electronic health record–based alerts had the lowest reported usability, suggesting EHR vendors should focus their efforts on improving alerts. Electronic health record efficiency strategies were broadly adopted, but only physicians with highly usable EHRs realized gains in EHR satisfaction from using these strategies, suggesting that EHR burden-reduction interventions are likely to have heterogenous associations across physicians with different EHRs.

## Key Points

**Question:**

How is the usability of different electronic health record (EHR) functions associated with physicians’ overall satisfaction with their EHR?

**Findings:**

This survey study of 2067 family physicians found that there was significant variation in usability across EHR functions, with alerts receiving the lowest score. Greater usability was associated with higher EHR satisfaction, and efficiency strategies were associated with improved satisfaction only for physicians with highly usable EHRs.

**Meaning:**

This study suggests that overall EHR usability and satisfaction vary greatly and that the benefits of EHR efficiency strategies are likely to be heterogenous across physicians depending on the usability of their EHR.

## Teaser

This survey study measures physician satisfaction with their electronic health record and its usability and evaluates the association of electronic health record usability with satisfaction and burnout, as well as the moderating association of electronic health record efficiency strategies.

## Introduction

Perhaps the most dramatic change to primary care over the past decade is the proportion of work mediated by electronic health records (EHRs).^1,2^ This “desktop medicine” includes patient history review, documentation, quality measure management, and responding to inbox messages—consuming nearly 50% of clinic time^3^ and frequently extending outside of clinic hours.^4^ Primary care physicians (PCPs) face the highest EHR burden, as they spend the most time using the EHR, receive the most inbox messages, and spend the most amount of after-hours time using the EHR,^5,6^ which contributes to burnout, turnover, and lower-quality care.^7,8,9,10^ Reducing EHR burden is a national policy priority as well as a key target for care delivery organizations across the country.^11,12^

Electronic health record system vendors, health systems, and PCPs have responded with an array of potential solutions. Vendors have focused on improving the usability of their software, including documentation, information presentation, and alerts.^13,14,15,16,17^ However, these efforts have not been guided by a robust understanding of which specific aspects of EHRs are associated with poor satisfaction for physicians. Evidence also suggests that poor usability is not the sole factor associated with EHR burden—notably, US physicians spend more time working in the EHR and write longer notes compared with their non-US peers using the same software, suggesting that sociotechnical factors, such as workflows, policy, reimbursement, and organizational support, are associated with EHR burden.^18,19^ To that end, care delivery organizations have deployed a number of EHR efficiency strategies, such as templated text documentation tools and team-based documentation workflows using scribes or other staff members. To date, there is limited generalizable evidence regarding the ability of these efficiency strategies to improve physician satisfaction with their EHR or overcome dissatisfaction with poor usability,^20,21,22,23^ making it difficult to assess what role these strategies should play in efforts to reduce EHR burden. It may be that physicians with significant support from teams or who are able to overcome usability challenges feel more satisfied with their EHR. If not, it suggests the need to develop new strategies to reduce EHR burden.

To inform national efforts to reduce EHR burden being pursued by multiple stakeholders, we used a national survey of US family physicians to address 3 research questions. First, to inform vendor efforts, how do PCPs rate satisfaction with their EHR and usability across different EHR functions, and what functions are rated as having the worst usability? Second, to inform health systems and practicing physicians, what team-based or technology-based efficiency strategies do PCPs use to optimize their EHR work, and how do they rate the effectiveness of those interventions at reducing EHR time? Third, how do those team-based and technology-based efficiency strategies moderate the association between usability and physician well-being outcomes, including EHR satisfaction and burnout? Results from our study have implications for policymakers, vendors, health systems, and physicians interested in addressing EHR burden, physician well-being, and burnout.

## Methods

### Survey and Data

We used data from the American Board of Family Medicine (ABFM) Continuous Certification Questionnaire, a mandatory part of extending certification as a family physician for those who sought recertification in 2022 and reported providing direct patient care. This ensured the survey had a 100% response rate among physicians who were continuing their certification in 2022, and questions could not be skipped. This study was approved by the institutional review board at the University of California, San Francisco with a waiver for informed consent due to the use of secondary data for research. This study followed the American Association for Public Opinion Research (AAPOR) reporting guideline for survey-based research by reporting the sample size and population being sampled, response rate, verbatim text of survey questions, and steps taken for pretesting and validation.

For more than a decade the ABFM has assessed EHR adoption and meaningful use requirements, and in 2022, the survey added questions regarding physician experience with the EHR. These new questions were extensively pretested with family physicians for content validity, including ten 1-hour semistructured interviews with physicians to ensure that questions were relevant and clear. Each physician answers a set of mandatory questions, then is sequentially randomized into 2 sets of different modules. The section on EHR satisfaction used in this study was 1 of 2 modules in the first set and included 50% of the respondents. In the second set of modules, physicians are randomized to 1 of 5 modules with 20% probability, 1 of which included the burnout questions also included in this study. The survey became available December 12, 2021, and closed October 17, 2022.

### Measures

#### EHR Satisfaction

We measured overall EHR satisfaction using responses to the question “Overall, how satisfied are you with your current primary, outpatient EHR system?” Responses on a 5-point Likert scale ranged from 1 (very dissatisfied) to 5 (very satisfied.)

#### EHR Function Usability

We measured PCP perceptions of usability across EHR functions using responses to the question “How would you assess the following usability dimensions of your current primary EHR system?” across the following 6 functions: entering information, readability of information, amount of information presented on each screen, alignment with the PCP’s workflow or cognitive process, ease of finding relevant information, and usefulness of alerts, all using a 4-point scale with responses options of poor (scored as 1), fair (scored as 2), good (scored as 3), and excellent (scored as 4), as well as not applicable. We constructed dichotomous measures of usability, classifying each domain as having good usability if the respondent selected good or excellent. We then constructed a usability index for each respondent by assigning each value a score of 1 (for poor) through 4 (for excellent). We scored responses of not applicable as 0, with the logic that respondents chose not to use that function of the EHR (eg, disabling alerts completely). We then took the summation of all 6 domains of functionality to create a composite measure of overall usability, based on a Cronbach α of 0.92 indicating strong internal consistency across the scale. Finally, we created a standardized version of our usability index to a mean of 0 and an SD of 1 to facilitate interpretation in multivariable models.

#### EHR Efficiency Strategies

We measured adoption and effectiveness of 4 common strategies meant to optimize EHR documentation efficiency: scribes, support from other staff (eg, medical assistants or nurses), templated text (eg, SmartPhrases or dot-phrases), and voice recognition or transcription, using responses to the question “Please indicate whether you use any of the following resources to help reduce time that you spend documenting in the EHR” with the response options including “yes, and it leads to reduced time,” “yes, but it does not reduce time,” and “no.” Because strategies may be duplicative, we classified physicians as those using no strategies, 1 strategy, and 2 or more strategies.

### Burnout

We measured burnout using responses to the question “I feel burned out from my work,” with response options never, a few times a year or less, once a month or less, a few times a month, once a week, a few times per week, or every day. This single-item measure has been validated as consistent with the full Maslach Burnout Inventory for PCPs.^24^ We operationalized this item by scoring respondents from 0 (for never) to 6 (for every day.)

### Robustness

We conducted a variety of tests to ensure that our results were robust to model specification and variable construction. First, we created a model with our usability index as a 25-level factor variable to flexibly estimate the association between EHR satisfaction and overall usability across the distribution of the usability index. Second, for multivariable models with EHR satisfaction as the dependent variable, we created models specifying our dependent variable as physicians responding either very satisfied or somewhat satisfied, to ensure our results were not due to the subset of very satisfied respondents. We also used this alternative construction in models using burnout as the dependent variable and EHR satisfaction as the independent variable of interest. Third, for all measures using a dichotomous dependent variable, we also used logistic regression, and for models using burnout as a dependent variable, we also used an ordinal logistic regression, to ensure our results were robust across different estimators. Fourth, we conducted a robustness test dropping all responses of not applicable to our function usability measures rather than coding them as zero.

### Statistical Analysis

We used descriptive statistics to assess sample demographics as well as physician overall EHR satisfaction. We then characterized perceptions of usability across our 6 domains and the use of 4 EHR efficiency strategies.

We then created several multivariable linear models using ordinary least-squares regression to evaluate the association between EHR usability, satisfaction, and documentation efficiency tool use. We chose ordinary least-squares models over logistic regression to facilitate interpretability of the results, as coefficients can be interpreted simply as the marginal association of the independent variable with the outcome.^25^ First, we evaluated the association between a dichotomous measure of whether a PCP responded that they were very satisfied with their EHR as the dependent variable and each functionality domain of EHR usability defined as a binary variable with respondents who selected good or excellent usability for that function compared with any other response as our independent variables of interest, including controls for EHR vendor, rurality, practice size, organization type, physician gender, value-based payment participation, and efficiency tool use with heteroskedasticity robust SEs.

Then, to evaluate the moderating association of strategies to optimize documentation efficiency with EHR satisfaction, we used another multivariable linear regression model with the same dichotomous dependent variable of physicians very satisfied with their EHR, while our independent variable of interest was an interaction term between the standardized usability index and the number of documentation efficiency strategies the physician responded that they had adopted and had saved them time (either 0, 1, or ≥2), in addition to the main associations of usability and efficiency strategies. We once again included controls for EHR vendor, rurality, practice size, organization type, physician gender, value-based payment participation, and each individual efficiency tool with heteroskedasticity-robust SEs. Then, to assess how the association between EHR satisfaction and efficiency strategy adoption varies across high-usability and low-usability EHRs, we used postestimation margins to compare associations between high (1 SD above the mean) and low (1 SD below the mean) usability across no, 1, and 2 or more efficiency strategies.

Finally, we evaluated the association between physician burnout and EHR satisfaction with a multivariable linear model with physician burnout frequency, scored as 0 through 6, as our dependent variable and a dichotomous measure of PCPs who reported they were very satisfied with their EHR as our independent variable of interest, again including controls for EHR vendor, rurality, practice size, organization type, physician gender, and value-based payment participation. All analyses were conducted using Stata, version 17.0 (StataCorp LLC), with 2-sided tests indicating statistical significance at *P* < .05.

## Results

### Sample Characteristics

Our analytic sample included 2067 family physicians in 2022; 431 (20.9%) were also administered the module on burnout. Physicians included 1051 men (50.9%) and 1016 women (49.2%), with 1246 (60.3%) younger than 50 years of age; 695 (34.3%) were located in the South, 570 (28.1%) in the West, 467 (23.0%) in the Midwest, and 297 (14.6%) in the Northeast (eTable 1 in Supplement 1). They practiced primarily in urban areas (1729 [86.0%]), at practice types including health systems (744 [36.0%]), independent practices (564 [27.3%]), federally qualified health centers or rural health centers (203 [9.8%]), academic centers (154 [7.5%]), and federal health systems such as the Veterans Health Administration (82 [4.0%]), with 320 (15.5%) responding that they practiced in another setting not listed. Most respondents worked in a practice with 2 to 5 clinicians (675 [32.7%]), followed by 6 to 20 clinicians (649 [31.4%]), more than 20 clinicians (558 [27.0%]), and finally solo practice (185 [9.0%]). A total of 1648 respondents (77.9%) practiced in a primary care clinic only, with 467 (22.1%) in a multispecialty practice. The most common EHR vendor was Epic (801 [38.8%]), followed by eClinical Works (209 [10.1%]), athenahealth (19 [9.3%]), and Cerner (163 [7.9%]). A total of 1386 respondents (67.1%) participated in some form of value-based care program.

### EHR Satisfaction and Usability

**Figure 1.**
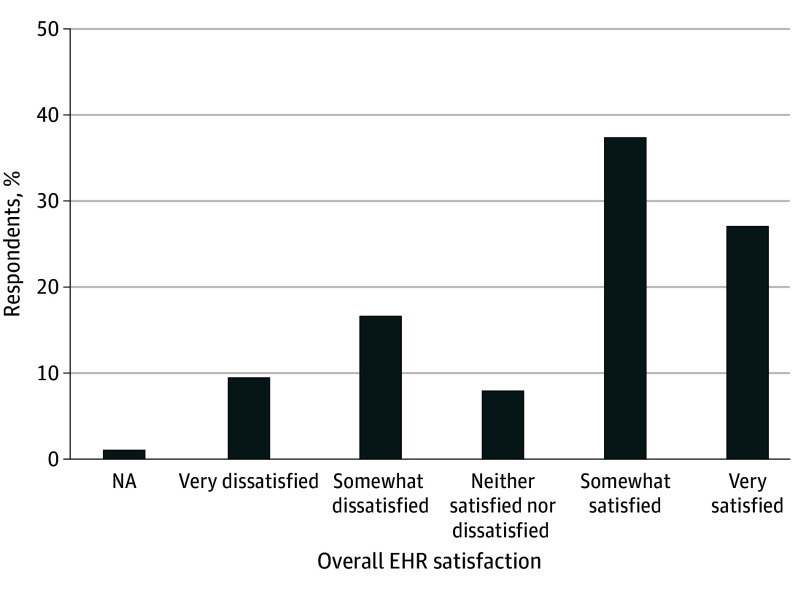
Physician Satisfaction With Their Electronic Health Record (EHR) NA indicates not applicable.

Most physicians were somewhat satisfied (775 [37.5%]) or very satisfied (562 [27.2%]) with their EHR, followed by somewhat dissatisfied (346 [16.7%]), very dissatisfied (198 [9.6%]), and neither satisfied nor dissatisfied (165 [8.0%]), with 21 (1.0%) selecting not applicable (Figure 1).

**Figure 2.**
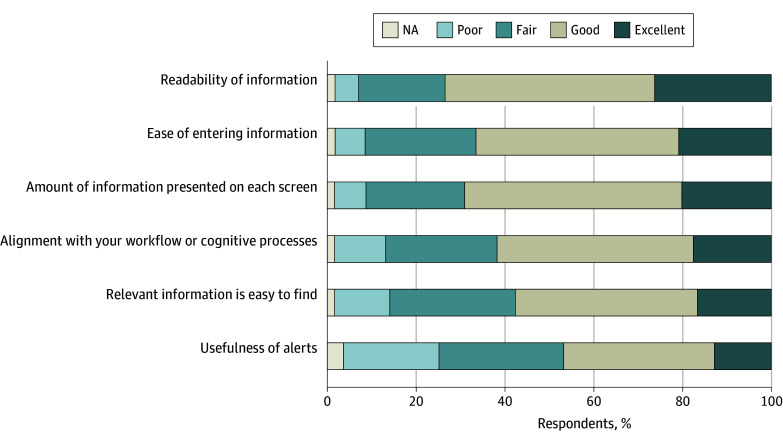
Electronic Health Record Usability by Function NA indicates not applicable.

For all domains of usability, the most common response was “good.” For readability of information, 976 physicians (47.2%) rated good while 543 (26.3%) rated excellent. For ease of entering information, 942 (45.6%) rated good and 429 (20.8%) reported excellent, while for usefulness of alerts, 702 (34.0%) rated good and 262 (12.7%) rated excellent (Figure 2).

### EHR Documentation Efficiency Strategy

**Figure 3.**
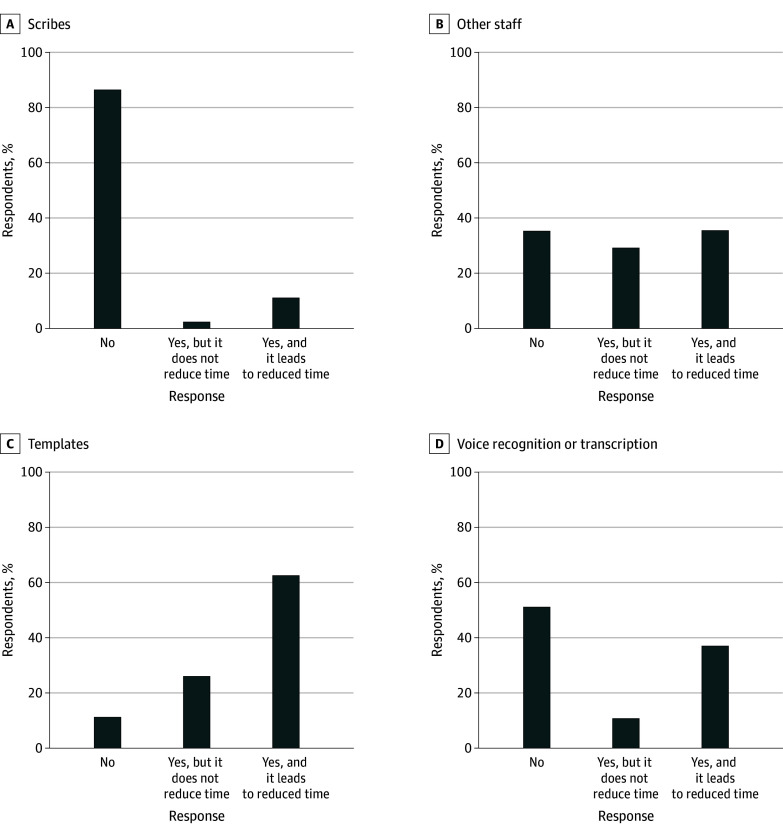
Physician Use of Efficiency Strategies and Team-Based Support to Reduce Electronic Health Record Burden

A total of 229 physicians (11.1%) in our sample reported using scribes and that they reduced EHR time, while 52 (2.5%) reported using scribes but said they did not reduce time in the EHR; 733 physicians (35.5%) reported using other staff for documentation and that this reduced EHR time, while 603 (29.2%) reported using other staff members but said they did not reduce EHR time. A total of 1289 physicians (62.4%) used EHR documentation templates and said they reduced EHR time, while 542 (26.2%) used templates but said they did not reduce EHR time. Finally, 771 physicians (37.3%) used voice recognition or transcription and said they reduced EHR time, while 236 (11.4%) reported using it but without a reduction in EHR time (Figure 3). Scribes were therefore perceived as the most effective at reducing EHR time (229 physicians [81.5%] who reported using scribes found they reduced EHR time), followed by voice recognition or transcription (771 [76.6%]), templated text (1289 [70.4%]), and finally other staff members (733 [54.9%]).

### Association Between EHR Satisfaction, Usability, and Burnout

In multivariable models, we found that that having good or excellent usability for entering data was associated with a 9–percentage point increase in the probability of a physician being very satisfied with their EHR (β = 0.09 [95% CI, 0.05-0.14]; *P* < .001). Alignment with workflow or cognitive processes (β = 0.11 [95% CI, 0.06-0.16]; *P* < .001), ease of finding relevant information (β = 0.14 [95% CI, 0.09-0.19]; *P* < .001), and usefulness of alerts (β = 0.11 [95% CI, 0.06-0.16]; *P* < .001) were all significantly associated with increased likelihood of EHR satisfaction, while usability of functions relating to how physicians consume information from the EHR, such as readability of information and amount of information presented on each screen, were not associated with EHR satisfaction (Table). We found similar results in our robustness tests (eTables 2-6 and the eFigure in Supplement 1).

**Table.** Associations of EHR Function Usability With EHR Satisfaction and Burnout

Model^a^	Coefficient (95% CI)	*P* value
Model 1. Dependent variable: EHR satisfaction		
Usability rating		
Entering data	0.09 (0.05 to 0.14)	<.001
Readability of information	0.01 (−0.03 to 0.06)	.56
Amount of information presented on each screen	−0.02 (−0.06 to 0.03)	.45
Alignment with your workflow or cognitive process	0.11 (0.06 to 0.16)	<.001
Relevant information is easy to find	0.14 (0.09 to 0.19)	<.001
Usefulness of alerts	0.11 (0.06 to 0.16)	<.001
Model 2. Dependent variable: frequency of physician burnout		
Overall EHR satisfaction		
Very dissatisfied, somewhat dissatisfied, neutral, or somewhat satisfied	[Reference]	
Very satisfied	−0.64 (−1.06 to −0.22)	<.001

Abbreviation: EHR, electronic health record.

^a^ Dependent variable for model 1 is a dichotomous measure of EHR satisfaction comparing physicians who respond that they are very satisfied with all other responses. Dependent variable for model 2 is burnout frequency scored from never (0) to every day (6), where a negative coefficient corresponds to less-frequent burnout. All models include controls for EHR vendor, rurality, practice size, organization type, physician gender, and value-based payment participation (not shown). Model 1 includes controls for efficiency of tool use (not shown).

In our multivariable models examining the association between our measure of EHR satisfaction and burnout, we found that being very satisfied with the EHR was associated with reduced burnout compared with physicians with any other level of EHR satisfaction (β = −0.64 [95% CI, −1.06 to −0.22]; *P* < .001) (Table). This corresponds to a roughly 18.8% lower burnout score. We found similar results in our robustness test using ordinal logistic regression (eTable 7 in Supplement 1).

### Moderation Analysis

**Figure 4.**
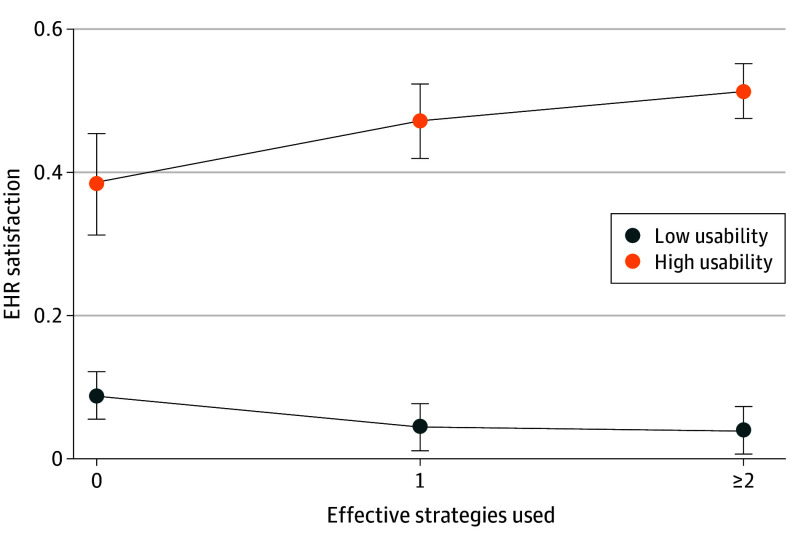
Moderation Analysis Between Usability, Burden-Reduction Strategies, and Satisfaction Results from multivariable regression model including controls for electronic health record (EHR) vendor, rurality, organization type, value-based payment participation, organization size, physician age, and physician gender. High usability indicates the respondent is 1 SD above the mean, while low usability indicates 1 SD below the mean in our standardized usability index. Error bars indicate 95% CIs from regression postestimation calculations.

In moderation analysis using our standardized usability index, we found that usability alone remained associated with EHR satisfaction, with physicians with highly usable EHRs (1 SD above the mean) 14.7 percentage points (95% CI, 11.0-18.4 percentage points) more likely to be very satisfied with their EHR (*P* < .001), while physicians using 1 (β = 0.02 [95% CI, –0.03 to 0.07]; *P* = .37) or 2 or more (β = 0.04 [95% CI, –0.01 to 0.09]; *P* = .10) efficiency strategies were not significantly more likely to report being very satisfied compared with physicians using no strategies. In our estimated margins comparing physicians with highly usable to low-usability EHRs across the use of 0, 1, or 2 or more efficiency strategies, we found that physicians with highly usable EHRs (1 SD above the mean, equivalent to a score of 21 on our 24-point usability scale) who used no efficiency strategies had a 38.5% chance (95% CI, 31.4%-45.5%) of being very satisfied with their EHR, those who used 1 efficiency strategy had a 47.4% chance (95% CI, 42.2%-52.6%), and those who used 2 or more efficiency strategies had a 51.4% chance (95% CI, 47.6%-55.2%) (Figure 4). However, the trend ran in the opposite direction for low-usability EHRs (1 SD below the mean, equivalent to a score of 11 on our 24-point usability scale)—physicians using no efficiency strategies had a 9.1% probability (95% CI, 5.7%-12.4%) of being very satisfied with their EHR, while those using 1 efficiency strategy (4.7% [95% CI, 10.4%-8.0%]) and 2 or more efficiency strategies (4.2% [95% CI, 0.8%-7.5%]) had a lower probability of being very satisfied with their EHR, showing that only physicians with highly usable EHRs realized gains in EHR satisfaction from efficiency strategies.

## Discussion

In a national survey with a 100% response rate, fewer than 30% of family physicians were very satisfied with their EHR, and more than one-fourth reported dissatisfaction. There was significant variation in usability across domains of the EHR, where most respondents reported that the ease of entering information and readability of information was good or excellent, while usefulness of EHR-based alerts was rated much lower. A small majority of family physicians used EHR templates to improve efficiency, but most do not have access to other efficiency strategies—fewer than 15% used scribes, which was rated as the most effective strategy to reduce documentation time. Although greater usability of most EHR functions, especially alignment with the physicians’ workflow or cognitive processes, usefulness of alerts, and ease of finding relevant information, were associated with greater EHR satisfaction, the use of EHR efficiency strategies improved satisfaction only for physicians with highly usable EHRs. Finally, we found physicians who responded that they were very satisfied with their EHR also reported significantly lower burnout.

We found that only one-fourth of family physicians were very satisfied with their EHR, while another one-fourth reported being somewhat dissatisfied or very dissatisfied, numbers consistent with other national surveys of physician EHR satisfaction.^4^ This finding is notable given the study’s 100% survey response rate, compared with much lower response rates to voluntary surveys, suggesting that physician dissatisfaction with the EHR is not due to response bias. Our results specifically suggest starting with addressing alerts, as they received the lowest usability scores. Although alerts have been shown to have important benefits (particularly reducing medication errors),^26,27^ research suggests that they are often poorly configured, have high dismissal rates, and have been associated with “alert fatigue” that harms physician well-being and patient outcomes.^16,17,28,29^ The focus of most alert-based research has been in the inpatient setting, where studies often find poor performance and a high level of “nuisance” alerts.^30,31^ Office-based physician alerts are relatively understudied and less sophisticated in comparison.^32^ A growing literature suggests that alerts can be improved through collaborative efforts between clinicians and informaticists,^33^ and recent policy action has added requirements for EHR vendors to support a feedback loop for decision support alert functionality.^34^ Although documentation makes up the plurality of time spent in the EHR and is the focus of several burden-reduction initiatives,^5,12^ fewer than 7% of physicians reported that the ease of entering information was poor, suggesting that it may not be usability challenges that are associated with documentation burden.

Many family physicians used at least 1 EHR documentation efficiency strategy, with the most common being documentation templates, which are low cost and may be included in default EHR builds. Templates, despite being broadly adopted, were far from the most effective strategies, adding evidence to research suggesting that while some use of templated text reduces documentation time, overreliance may increase EHR burden as physicians document to the template, rather than each patient’s specific needs.^21,22^ Although scribes were reported as the most effective documentation efficiency strategy among physicians who reported using them, documentation by other staff members was the least effective. It may be that while offloading documentation to staff reduces EHR burden, effective implementation requires a dedicated role and accompanying workflow adaptations, rather than relying on other staff on an ad hoc basis.

Despite many physicians using at least 1 effective efficiency strategy, our moderation analysis found that while usability was associated with overall EHR satisfaction, the benefits of these efficiency strategies were realized only by physicians with highly usable EHRs. Understanding this association is critical to informing future efforts to address EHR burden. It may simply be that at low levels, usability dominates physician perception of their EHR so strongly that effective team-based or technology-based efficiency strategies are not able to overcome poor software usability. If this is the case, resources to improve documentation efficiency are unlikely to help physicians with low-usability EHRs. Given that the usability of entering information is frequently rated higher than functions such as finding information or alerts, it is likely that documentation-focused efficiency strategies such as scribes or templated text are unable to address the salient pain points of low-usability EHRs. Instead, technology tools that may be on the horizon, such as artificial intelligence that summarizes bloated notes or improved data standards to ensure patient information is available in a standardized format, may be necessary to improve EHR satisfaction for these physicians. Conversely, for physicians with highly usable EHRs, strategies to reduce documentation time are associated with EHR satisfaction, suggesting that investments in these team and technology interventions are worthwhile. Our results therefore suggest that the effects of popular EHR burden-reduction interventions are likely to be heterogenous across users with different levels of EHR usability. Physicians, EHR vendors, and health system leaders should design burden-reduction efforts with these results in mind. The association between high EHR satisfaction and burnout highlights the potential for renewed policymaker and vendor attention to EHR optimization to contribute to broader efforts to address persistent and increasing rates of physician burnout in primary care.^35^

### Limitations

Our study has some limitations, including that results from our cross-sectional descriptive analysis precludes causal inference. Second, our survey does not assess granular details, such as the intensity of documentation efficiency strategies. For example, there is likely variation across physicians using staff for documentation (ie, the staff member performing the task and the degree of training they receive) that we do not observe. To address this concern, our measure of efficiency strategy use included only physicians who reported that those efficiency strategies reduced EHR documentation time, but there is likely variation in implementation of each strategy. Future research should explore what specific team, technology, and contextual factors facilitate successful implementation of each EHR documentation efficiency strategy. Our study assesses only the association between EHR satisfaction and burnout, but many other factors, including work environment and feeling valued, are known to be associated with burnout, and we were unable to fully capture the spectrum of possible antecedents to burnout. Finally, the survey-based nature of our study relied on family physicians’ self-reported data that we were unable to independently verify. However, our high-level results are similar to other survey-based estimates of PCP EHR use.^4^

## Conclusions

This unique national survey with a 100% response rate found that only one-fourth of physicians were very satisfied with their EHR, while a substantial minority were dissatisfied, and usability was correlated with overall EHR satisfaction. Although most physicians reported using some type of efficiency strategy, such as scribes, templated text, or voice recognition to reduce EHR documentation time, gains in EHR satisfaction were realized only by physicians with high-usability EHRs, suggesting that addressing physician EHR burden requires a targeted approach to address the EHR needs of the specific physician.

## Notes

Supplement 1. **eTable 1.** Sample Demographic Characteristics **eTable 2.** Multivariable Models Using Usability Index Factor Variable as Independent Variable of Interest **eTable 3.** Multivariable Models on Association Between EHR Function Usability and EHR Satisfaction Using a “Very” or “Somewhat” Satisfied Dependent Variable **eTable 4.** Multivariable Model on Association Between EHR Satisfaction and Burnout Using a “Very” or “Somewhat” Satisfied With EHR Independent Variable **eTable 5.** Multivariable Models on Association Between EHR Function Usability and EHR Satisfaction Using Logistic Regression **eTable 6.** Associations of EHR Function Usability With EHR Satisfaction and Burnout Without “Not Applicable” Respondents **eFigure.** Moderation Analysis Between Usability, Burden Reduction Strategies, and Satisfaction Without “Not Applicable” Responses **eTable 7.** Ordinal Logistic Regression of the Relationship Between Burnout and EHR Satisfaction

Supplement 2.
Data Sharing Statement
