# Supplementary material for: Electronic Health Record Usability, Satisfaction, and Burnout for Family Physicians
Source: JAMA Netw Open. 2024 Aug 29;7(8):e2426956. doi: 10.1001/jamanetworkopen.2024.26956 (PMC11362862; doi:10.1001/jamanetworkopen.2024.26956)
Supplement: Supplement 1. — eTable 1. Sample Demographic Characteristics eTable 2. Multivariable Models Using Usability Index Factor Variable as Independent Variable of Interest eTable 3. Multivariable Models on Association Between EHR Function Usability and EHR Satisfaction Using a “Very” or “Somewhat” Satisfied Dependent Variable eTable 4. Multivariable Model on Association Between EHR Satisfaction and Burnout Using a “Very” or “Somewhat” Satisfied With EHR Independent Variable eTable 5. Multivariable Models on Association Between EHR Function Usability and EHR Satisfaction Using Logistic Regression eTable 6. Associations of EHR Function Usability With EHR Satisfaction and Burnout Without “Not Applicable” Respondents eFigure. Moderation Analysis Between Usability, Burden Reduction Strategies, and Satisfaction Without “Not Applicable” Responses eTable 7. Ordinal Logistic Regression of the Relationship Between Burnout and EHR Satisfaction [file jamanetwopen-e2426956-s001.pdf]

Supplementary Online Content

Holmgren AJ, Hendrix N, Maisel N, et al. Electronic health record usability, satisfaction, and burnout for family physicians. *JAMA Netw Open.* 2024;7(8):e2426956. doi:10.1001/jamanetworkopen.2024.26956

eTable 1. Sample Demographic Characteristics
eTable 2. Multivariable Models Using Usability Index Factor Variable as Independent Variable of Interest
eTable 3. Multivariable Models on Association Between EHR Function Usability and EHR Satisfaction Using a "Very" or "Somewhat" Satisfied Dependent Variable
eTable 4. Multivariable Model on Association Between EHR Satisfaction and Burnout Using a "Very" or "Somewhat" Satisfied With EHR Independent Variable
eTable 5. Multivariable Models on Association Between EHR Function Usability and EHR Satisfaction Using Logistic Regression
eTable 6. Associations of EHR Function Usability With EHR Satisfaction and Burnout Without "Not Applicable" Respondents
eFigure. Moderation Analysis Between Usability, Burden Reduction Strategies, and Satisfaction Without "Not Applicable" Responses
eTable 7. Ordinal Logistic Regression of the Relationship Between Burnout and EHR Satisfaction

This supplementary material has been provided by the authors to give readers additional information about their work.

eTable 1. Sample Demographic Characteristics

**Gender**	N	%
Female	1,016	49.15
Male	1,051	50.85
**Age**		
50+	821	39.72
<50	1,246	60.28
**US Census Region**		
Midwest	467	23.02
Northeast	297	14.64
South	695	34.25
West	570	28.09
**Rurality**		
Rural	282	14.02
Urban	1,729	85.98
**Practice Setting**		
Academic	154	7.45
FQHC/RHC	203	9.82
Federal	82	3.97
Health System	744	35.99
Independent	564	27.29
Other	320	15.48
**Practice Size**		
Solo practice	185	8.95
2-5 Providers	675	32.66
6-20 Providers	649	31.4
>20 Providers	558	27.00
**Practice Specialty**		
Primary Care Only	1,648	77.92
Multi-Specialty	467	22.08
**EHR Vendor**		
Epic	801	38.75
eClinical Works	209	10.11
athenahealth	192	9.29
Cerner	163	7.89
Allscripts	105	5.08
NextGen	82	3.97
Unknown	45	2.18
Practice Fusion	37	1.79
Greenway	33	1.60

© 2024 Holmgren AJ et al. *JAMA Network Open.*

e-MDs	14	0.68
Other	386	18.67
**Participation in Any Value-Based Care Program**		
Don't Know	412	19.93
No	269	13.01
Yes	1,386	67.05

© 2024 Holmgren AJ et al. *JAMA Network Open.*

eTable 2. Multivariable Models Using Usability Index Factor Variable as Independent Variable of Interest

DV: Very Satisfied with EHR	Coefficient	p-value	[95% conf.	interval]
Usability Index (0-24)				
0	Ref.			
1	0.99	<0.001	0.88	1.10
4	1.08	<0.001	0.97	1.19
6	-0.02	0.666	-0.11	0.07
7	0.02	0.723	-0.11	0.16
8	-0.02	0.639	-0.10	0.06
9	-0.02	0.688	-0.11	0.08
10	-0.03	0.557	-0.11	0.06
11	-0.02	0.689	-0.11	0.07
12	0.04	0.373	-0.05	0.13
13	0.01	0.913	-0.09	0.10
14	0.05	0.33	-0.05	0.14
15	0.05	0.33	-0.05	0.14
16	0.11	0.045	0.00	0.21
17	0.22	<0.001	0.11	0.33
18	0.29	<0.001	0.20	0.39
19	0.38	<0.001	0.25	0.52
20	0.43	<0.001	0.29	0.56
21	0.43	<0.001	0.28	0.59
22	0.50	<0.001	0.35	0.66
23	0.61	<0.001	0.46	0.77
24	0.76	<0.001	0.66	0.86
Efficiency Strategy: Scribes				
No	Ref.			
Yes, effectively	-0.01	0.867	-0.11	0.09
Yes, does not reduce time	-0.02	0.403	-0.08	0.03
Efficiency Strategy: Other Staff for Documentation				
No	Ref.			
Yes, effectively	-0.02	0.331	-0.06	0.02
Yes, does not reduce time	-0.01	0.569	-0.06	0.03
Efficiency Strategy: Templates				
No	Ref.			
Yes, effectively	-0.04	0.143	-0.10	0.01
Yes, does not reduce time	-0.01	0.603	-0.07	0.04

© 2024 Holmgren AJ et al. *JAMA Network Open.*

Efficiency Strategy: Voice Recognition / Transcription				
No	Ref.			
Yes, effectively	0.01	0.677	-0.04	0.06
Yes, does not reduce time	0.04	0.062	0.00	0.07
EHR Vendor				
Unknown	Ref.			
Allscripts	0.01	0.896	-0.11	0.13
Cerner	-0.03	0.55	-0.15	0.08
Epic	0.10	0.073	-0.01	0.21
Greenway	0.03	0.7	-0.13	0.19
NextGen	0.12	0.079	-0.01	0.25
Other	0.06	0.295	-0.05	0.17
Practice Fusion	0.03	0.741	-0.14	0.19
athenahealth	0.11	0.072	-0.01	0.23
e-MDs	-0.01	0.907	-0.24	0.21
eClinical Works	0.05	0.421	-0.07	0.16
Rurality				
Rural	Ref.			
Urban	0.00	0.87	-0.04	0.05
Practice Type				
Other	Ref.			
Academic	0.05	0.137	-0.02	0.13
FQHC/RHC	0.07	0.028	0.01	0.14
Federal	0.04	0.345	-0.04	0.11
Health System	0.04	0.115	-0.01	0.09
Independent	0.03	0.244	-0.02	0.09
VBP				
Don't Know	Ref.			
No	-0.04	0.183	-0.10	0.02
Yes	0.00	0.868	-0.05	0.04
Size				
2-5 Providers	Ref.			
6-20 Providers	-0.02	0.395	-0.06	0.02
>20 Providers	-0.05	0.023	-0.10	-0.01
Solo practice	0.00	0.969	-0.07	0.07

© 2024 Holmgren AJ et al. *JAMA Network Open.*

Gender				
Female	Ref.			
Male	0.00	0.967	-0.03	0.03
Age				
>50	Ref.			
<50	0.04	0.026	0.00	0.07

© 2024 Holmgren AJ et al. *JAMA Network Open.*

eTable 3. Multivariable Models on Association Between EHR Function Usability and EHR Satisfaction Using a "Very" or "Somewhat" Satisfied Dependent Variable

DV: Somewhat or Very Satisfied with EHR	Coefficient	p-value	[95% conf.	interval]
Usability Rating				
Entering Data	0.10	0.00	0.07	0.14
Readability of information	0.00	0.99	-0.04	0.03
Amount of information presented on each screen	-0.01	0.74	-0.04	0.03
Alignment with your workflow/cognitive process	0.12	0.00	0.08	0.15
Relevant information is easy to find	0.05	0.00	0.02	0.09
Usefulness of alerts	0.01	0.60	-0.02	0.03
Efficiency Strategy: Scribes				
No	Ref.			
Yes, effectively	0.00	0.96	-0.12	0.12
Yes, does not reduce time	-0.02	0.61	-0.07	0.04
Efficiency Strategy: Other Staff for Documentation				
No	Ref.			
Yes, effectively	-0.01	0.58	-0.06	0.03
Yes, does not reduce time	-0.02	0.32	-0.07	0.02
Efficiency Strategy: Templates				
No	Ref.			
Yes, effectively	-0.02	0.48	-0.09	0.04
Yes, does not reduce time	-0.02	0.44	-0.09	0.04
Efficiency Strategy: Voice Recognition / Transcription				
No	Ref.			
Yes, effectively	-0.05	0.15	-0.11	0.02
Yes, does not reduce time	0.00	0.99	-0.04	0.04
EHR Vendor				
Unknown	Ref.			
Allscripts	0.01	0.94	-0.14	0.15
Cerner	-0.08	0.29	-0.22	0.06
Epic	0.07	0.30	-0.06	0.20
Greenway	-0.06	0.56	-0.24	0.13
NextGen	0.05	0.53	-0.10	0.20
Other	-0.04	0.59	-0.17	0.09
Practice Fusion	-0.14	0.18	-0.34	0.07

© 2024 Holmgren AJ et al. *JAMA Network Open.*

athenahealth	-0.02	0.77	-0.16	0.12
e-MDs	0.08	0.47	-0.14	0.30
eClinical Works	0.01	0.86	-0.12	0.15
Rurality				
Rural	Ref.			
Urban	0.00	0.88	-0.06	0.05
Practice Type				
Other	Ref.			
Academic	0.06	0.17	-0.03	0.14
FQHC/RHC	0.05	0.21	-0.03	0.12
Federal	0.05	0.43	-0.07	0.16
Health System	0.04	0.22	-0.02	0.10
Independent	0.05	0.13	-0.02	0.12
VBP				
Don't Know	Ref.			
No	-0.02	0.61	-0.09	0.05
Yes	-0.01	0.71	-0.06	0.04
Size				
2-5 Providers	Ref.			
6-20 Providers	0.03	0.28	-0.02	0.07
>20 Providers	0.00	1.00	-0.05	0.05
Solo practice	0.11	0.01	0.03	0.18
Gender				
Female	Ref.			
Male	0.00	0.97	-0.04	0.04
Age				
>50	Ref.			
<50	0.06	0.00	0.02	0.10

© 2024 Holmgren AJ et al. *JAMA Network Open.*

eTable 4. Multivariable Model on Association Between EHR Satisfaction and Burnout Using a "Very" or "Somewhat" Satisfied With EHR Independent Variable

DV: Frequency of Burnout (0 - 6)	Coefficient	p-value	[95% conf.	interval]
EHR Satisfaction				
Somewhat or Very Satisfied	-0.54	0.00	-0.90	-0.18
EHR Vendor				
Unknown	Ref.			
Allscripts	1.65	0.02	0.28	3.02
Cerner	1.55	0.01	0.35	2.75
Epic	1.87	0.00	0.80	2.93
Greenway	1.54	0.02	0.28	2.80
NextGen	1.25	0.05	0.01	2.50
Other	1.16	0.04	0.05	2.27
Practice Fusion	3.09	0.00	1.78	4.40
athenahealth	1.47	0.02	0.28	2.66
e-MDs	2.57	0.09	-0.40	5.54
eClinical Works	1.93	0.00	0.86	3.01
Rurality				
Rural	Ref.			
Urban	-0.33	0.20	-0.82	0.17
Practice Type				
Other	Ref.			
Academic	-0.61	0.10	-1.36	0.13
FQHC/RHC	-0.20	0.58	-0.92	0.52
Federal	1.50	0.00	0.57	2.43
Health System	0.41	0.17	-0.17	1.00
Independent	0.27	0.41	-0.38	0.92
VBP				
Don't Know	Ref.			
No	0.29	0.39	-0.37	0.94
Yes	0.42	0.06	-0.02	0.87
Size				
2-5 Providers	Ref.			
6-20 Providers	0.21	0.37	-0.25	0.68
>20 Providers	0.25	0.29	-0.22	0.73

© 2024 Holmgren AJ et al. *JAMA Network Open.*

Solo practice	0.19	0.61	-0.52	0.89
Gender				
Female	Ref.			
Male	-0.43	0.02	-0.78	-0.08
Age				
>50	-0.23	0.23	-0.62	0.15
<50	0.06	0.00	0.02	0.10

© 2024 Holmgren AJ et al. *JAMA Network Open.*

eTable 5. Multivariable Models on Association Between EHR Function Usability and EHR Satisfaction Using Logistic Regression

Model 1. Individual Usability Functions

Dependent Variable: EHR Satisfaction	Odds Ratio	p-value	95%	CI
**Usability Rating**				
Entering Data	2.56	<0.001	1.71	3.85
Readability of information	1.35	0.21	0.84	2.16
Amount of information presented on each screen	1.02	0.90	0.68	1.55
Alignment with your workflow/cognitive process	2.47	<0.001	1.69	3.63
Relevant information is easy to find	2.61	<0.001	1.85	3.69
Usefulness of alerts	1.67	<0.001	1.27	2.20

© 2024 Holmgren AJ et al. *JAMA Network Open.*

eTable 6. Associations of EHR Function Usability With EHR Satisfaction and Burnout Without “Not Applicable” Respondents

Dependent Variable: EHR Satisfaction	Coefficient (95% Confidence Interval)	p-value
**Usability Rating**		
Entering Data	0.08 (0.04 - 0.13)	<0.001
Readability of information	0.02 (-0.03 - 0.06)	0.42
Amount of information presented on each screen	-0.02 (-0.07 - 0.03)	0.38
Alignment with your workflow/cognitive process	0.11 (0.06 - 0.16)	<0.001
Relevant information is easy to find	0.14 (0.09 - 0.19)	<0.001
Usefulness of alerts	0.11 (0.07 - 0.17)	<0.001

© 2024 Holmgren AJ et al. *JAMA Network Open.*

eFigure. Moderation Analysis Between Usability, Burden Reduction Strategies, and Satisfaction Without “Not Applicable” Responses

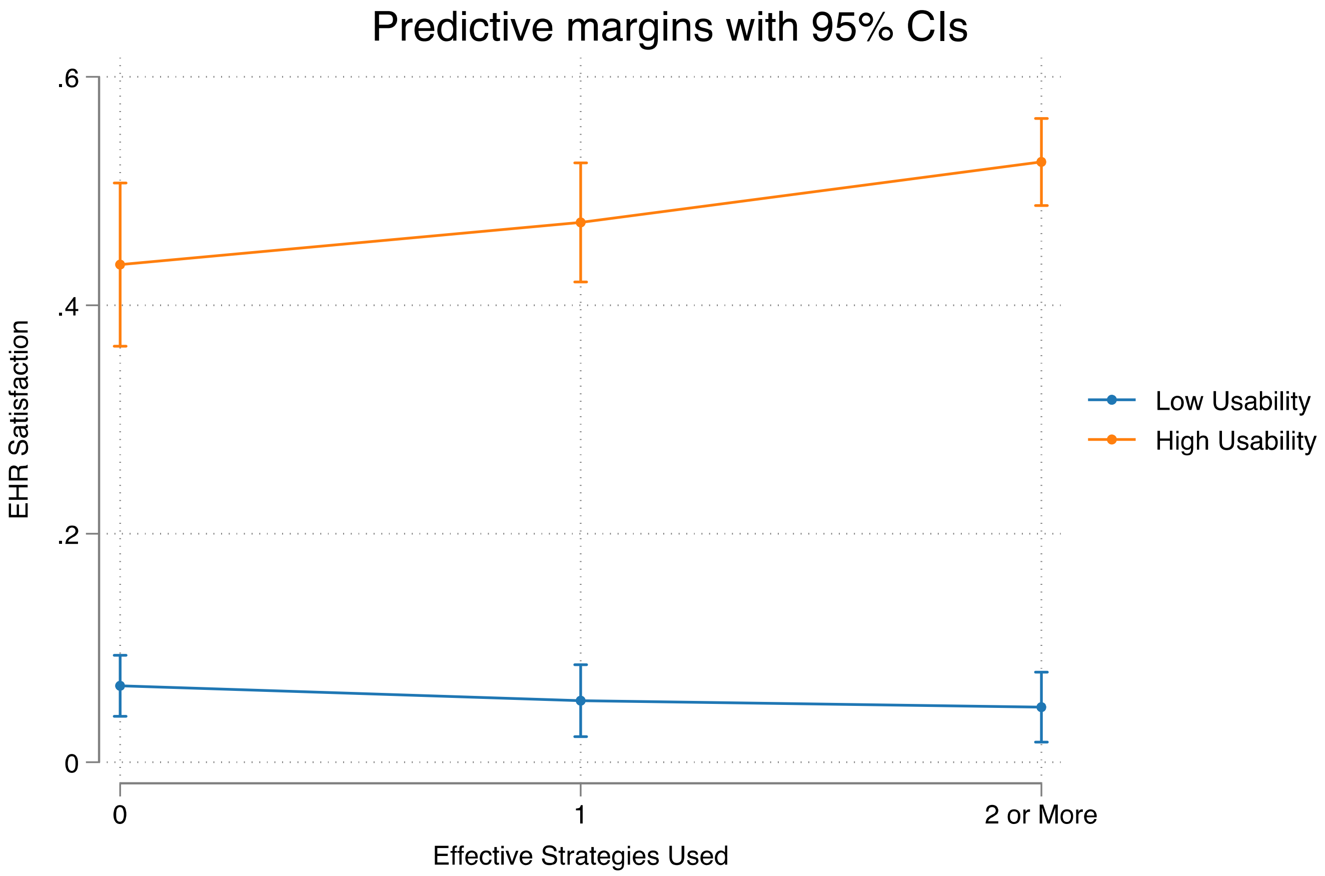

© 2024 Holmgren AJ et al. *JAMA Network Open.*

eTable 7. Ordinal Logistic Regression of the Relationship Between Burnout and EHR Satisfaction

Dependent Variable: Frequency of Physician Burnout	Odds Ratio (95% Confidence Interval)	p-value
**Overall EHR Satisfaction**		
Very Dissatisfied/Somewhat Dissatisfied/Neutral/Somewhat Satisfied	Ref	
Very Satisfied with EHR	0.56 (0.35 – 0.90)	0.02

© 2024 Holmgren AJ et al. *JAMA Network Open.*
