# Supplementary material for: Electronic Health Record Usability, Satisfaction, and Burnout for Family Physicians
Source: JAMA Netw Open. 2024 Aug 29;7(8):e2426956. doi: 10.1001/jamanetworkopen.2024.26956 (PMC11362862; doi:10.1001/jamanetworkopen.2024.26956)
Supplement: Supplement 2. — Data Sharing Statement [file jamanetwopen-e2426956-s002.pdf]

Data Sharing Statement

Holmgren. Electronic Health Record Usability, Satisfaction, and Burnout for Family Physicians. *JAMA Netw Open*. Published August 29, 2024. doi:10.1001/jamanetworkopen.2024.26956

Data
Data available: No

Additional Information
Explanation for why data not available: Data is proprietary to the American Board of Family Medicine.
